# Metagenomic sequencing of stool samples in Bangladeshi infants: virome association with poliovirus shedding after oral poliovirus vaccination

**DOI:** 10.1038/s41598-020-71791-4

**Published:** 2020-09-21

**Authors:** Susanna K. Tan, Andrea C. Granados, Jerome Bouquet, Yana Emmy Hoy-Schulz, Lauri Green, Scot Federman, Doug Stryke, Thomas D. Haggerty, Catherine Ley, Ming-Te Yeh, Kaniz Jannat, Yvonne A. Maldonado, Raul Andino, Julie Parsonnet, Charles Y. Chiu

**Affiliations:** 1 Division of Infectious Diseases, Department of Medicine, Stanford University School of Medicine, Stanford, CA USA; 2 Department of Laboratory Medicine, University of California, San Francisco, CA USA; 3 UCSF-Abbott Viral Diagnostics and Discovery Center, San Francisco, CA USA; 4 Department of Microbiology and Immunology, University of California, San Francisco, CA USA; 5 Environmental Intervention Unit, Infectious Disease Division, International Centre for Diarrheal Disease Research, Dhaka, Bangladesh; 6 Division of Infectious Diseases, Department of Pediatrics, Stanford University School of Medicine, Stanford, CA USA; 7 Department of Epidemiology and Population Health, Stanford University School of Medicine, Stanford, CA USA; 8 Division of Infectious Diseases, Department of Medicine, University of California, 185 Berry Street, Box #0134, San Francisco, CA 94107 USA

**Keywords:** Genomic analysis, Microbiology techniques, Sequencing, Medical research, Infectious diseases

## Abstract

The potential role of enteric viral infections and the developing infant virome in affecting immune responses to the oral poliovirus vaccine (OPV) is unknown. Here we performed viral metagenomic sequencing on 3 serially collected stool samples from 30 Bangladeshi infants following OPV vaccination and compared findings to stool samples from 16 age-matched infants in the United States (US). In 14 Bangladeshi infants, available post-vaccination serum samples were tested for polio-neutralizing antibodies. The abundance (p = 0.006) and richness (p = 0.013) of the eukaryotic virome increased with age and were higher than seen in age-matched US infants (p < 0.001). In contrast, phage diversity metrics remained stable and were similar to those in US infants. Non-poliovirus eukaryotic virus abundance (3.68 log_10_ vs. 2.25 log_10_, p = 0.002), particularly from potential viral pathogens (2.78log_10_ vs. 0.83log_10_, p = 0.002), and richness (p = 0.016) were inversely associated with poliovirus shedding. Following vaccination, 28.6% of 14 infants tested developed neutralizing antibodies to all three Sabin types and also exhibited higher rates of poliovirus shedding (p = 0.020). No vaccine-derived poliovirus variants were detected. These results reveal an inverse association between eukaryotic virome abundance and poliovirus shedding. Overall gut virome ecology and concurrent viral infections may impact oral vaccine responsiveness in Bangladeshi infants.

## Introduction

Oral vaccines are more effective in high-income countries than in low-income countries^1^. One hypothesis for this finding is potential blocking of effective immune responses by bacteria, phages and eukaryotic viruses that inhabit similar niches as live vaccine strains. Concurrent infection with non-polio enteroviruses has been previously reported to interfere with oral poliovirus vaccine (OPV) efficacy^2^. Concurrent administration of oral poliovirus and rotavirus vaccine has also been shown to reduce rotavirus vaccine immunogenicity^3,4^.

Most infections in children under five years old are caused by viruses. Our understanding of the pediatric virome, however, remains rudimentary. Metagenomic next-generation sequencing approaches have previously demonstrated that the virome in children is dynamic and that virus distributions are driven by a combination of maternal, geographic and environmental factors^5–7^.

In this study, we applied metagenomic virus sequencing to characterize the gastrointestinal virome in serially collected stool samples from infants from Bangladesh. All of the infants evaluated in this study completed the first two OPV vaccinations (out of 3 for the full series), with the first dose administered within the first 4 months of life. OPV consists of trivalent live-attenuated Sabin poliovirus that replicates at mucosal sites in the infant gastrointestinal tract and induces mucosal and systemic antibody production^1^. Here, we describe the infant virome in Bangladesh in comparison to that of US children and investigate the role of the virome on shedding of vaccine-associated poliovirus and the development of effective poliovirus neutralizing antibody responses.

## Methods

### Study population and sample collection

Stool samples for virome analysis were obtained from a phase I trial of probiotics in infants in Bangladesh, as previously described^8^. These infants all came from households of low socioeconomic status living in peri-urban slums. Infants were chosen from among the 160 babies in the trial based on the completeness of fecal sampling. Preference was given to those receiving the lowest dose of the combined lactobacillus and Bifidobacterium probiotic: five subjects were in the control arm (no probiotic), 12 were in the twice per month probiotic arm and 13 were in the weekly probiotic arm. Probiotics, consisting of *Lactobacillus reuteri* DSM 17,938 combined with *Bifidobacterium longdum subspecies infantis* 35,624, were given for one month.

Infants aged 4–12 weeks (mean age 8 weeks) were recruited from three vaccination clinics near the International Center for Diarrheal Disease Research, Bangladesh (icddr,b) in Dhaka between October 2013 and April 2014. All infants received at least the first 2 doses of the trivalent oral polio vaccination (OPV) that is administered 6, 10 and 14 weeks old; no infants were given OPV vaccine at birth. The dates of vaccination were documented by vaccination cards (> 90% of the time), or in rare instances, estimated from parents’ recollection. Other vaccines given at these timepoints included pentavalent (diphtheria, tetanus, pertussis, *Haemophilus influenzae* type B, and hepatitis B virus) and pneumococcal vaccines (Supplementary Table S1). Demographic and socioeconomic data were collected at enrollment. Health information for the infants in the study, including illness, gastrointestinal and respiratory symptoms and breastfeeding practices were collected at weekly intervals (Table 1).

**Table 1:** Characteristics of the Bangladeshi infants in the study.

Characteristic	Median (IQR) or N (%)
Age in weeks^a^	9.9 (9.6–10.9)
Weight Z-score^a^	− 0.76 (− 1.22 to − 0.26)
Height Z-score^a^	− 0.45 (− 1.29 to − 0.54)
Head circumference Z-score^a^	− 1.14 (− 1.92 to − 0.49)
Female	14 (47)
Born by cesarean section	7 (23)
Years of maternal education	5 (4–8)
Household size	5 (3–6)
**Household monthly income**	
< $100	7 (23)
$100–$150	12 (40)
> $150	11 (37)
**Diet**	
Exclusive breastfed	10 (30)
Partial breastfed (Supplementary feeding)	20 (60)
Cow’s milk	3 (6.7)
Water, formula, or baby cereal	20 (60)
Antibiotic exposure during study	4 (13.3)
**Illness symptoms during study**	
No symptoms	8 (26.6)
Fever	12 (40)
Respiratory (cough, congestion)	19 (63)
Gastrointestinal (vomiting, watery stool)	3 (10)

^a^At first stool sample.

*IQR* interquartile range.

The study was approved by the institutional review boards at both icddr,b (Protocol ID 13,022) and Stanford University (Protocol ID 25,487) and was registered on ClinicalTrials.gov (NCT01899378). Written informed consent was provided by parents or guardians. As part of their consent, subjects agreed to unspecified “advanced tests” of their stool samples “that may help us better understand the infections that your baby may have had”’; the virome analyses described here fall in this category. All samples were anonymized prior to virome analyses. All research was performed in accordance with guidelines and regulations on human subjects research established by the IRBs at UCSF and Stanford University, the National Institutes of Health, and the World Medical Association (WMA) Declaration of Helsinki.

**Figure 1:**
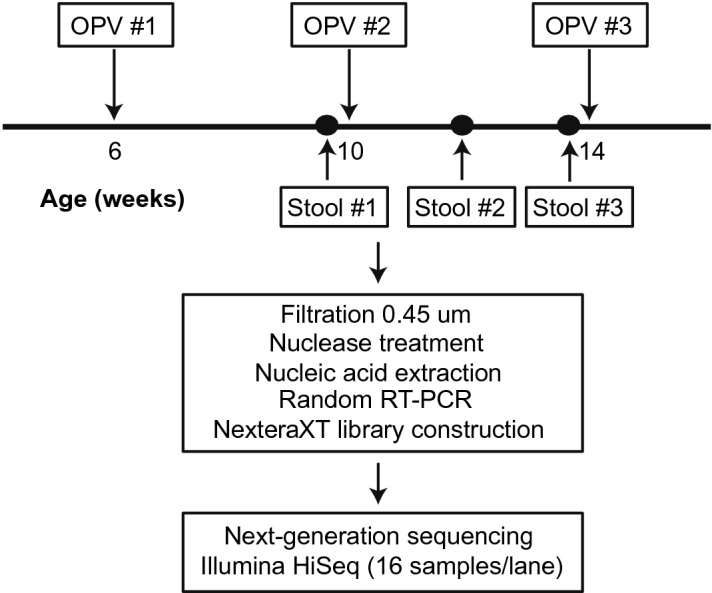
Overview of sample collection and virus metagenomic sequencing protocol. Abbreviations: OPV, oral poliovirus vaccine.

Viral metagenomic analysis was performed on three stool samples from 30 infants collected at 4 weeks after the first dose of OPV vaccine (prior to the second dose), 2 weeks after the second dose of OPV vaccine, and 4 weeks after the second dose of OPV vaccine (immediately prior to the third dose) (Fig. 1). Stool samples from infants prior to OPV vaccination were not available as infants had already received the first OPV vaccination at time of enrollment. For purposes of comparison, stool samples were also tested from age-matched infants in California, USA from the Stanford’s Outcome Research in Kids (STORK) cohort, a longitudinal study of the impact of the developing virome and pediatric infections on weight, growth and immune development in infants^9^. Specifically, we included 16 infants from the STORK cohort with available stool samples collected prior to 8 weeks of age and administration of rotavirus vaccine. The STORK study was approved by the Institutional Boards of Stanford University and the Santa Clara Valley Medical Center, and written informed consent was obtained from parents or guardians.

Stool samples from Bangladeshi and California infants were collected in sterile containers and processed in an identical fashion for virome analysis. For the Bangladeshi cohort, fresh stool samples collected in the field were placed on ice and then brought to the lab the same day on ice and frozen within 10 h of collection. For the California infants in the STORK cohort, fresh stool samples collected in the clinic were placed on ice packs and frozen within 24 h of collection. Frozen stool samples were stored at – 80 °C prior to processing.

### Nucleic acid extraction

Nucleic acid extraction of stool samples was performed as previously described^10^. Stool samples were diluted 20% in phosphate buffered saline (PBS) (1,500 μl) and centrifuged for 5 min at 10,000*g*, followed by filtration using a 0.45 μM filter and treatment using a nuclease cocktail of TURBO DNase (Invitrogen), Baseline Zero DNase (Ambion), Benzonase (Novagen) and RNase A (Roche) for 30 min at 37 °C. This procedure digests host cell and non-protected (“naked”) viral nucleic acids, while maintaining viral RNA in particles that are protected from the action of nucleases^11^. Nuclease activity was then immediately inactivated by addition of guanidium-thiocyanate containing lysis buffer (Qiagen), followed by total nucleic acid extraction of 400 μl of pretreated stool using the EZ1 Virus Mini Kit v2.0 (Qiagen). Extracts were eluted in 60 μl volume.

### Library preparation and viral metagenomic next-generation sequencing

Amplified cDNA was prepared using random nonamer primers attached to a primer linker sequence with 25 cycles of PCR amplification, as previously described^12,13^. Ultra-pure bovine serum albumin (BSA) (Ambion) was added to the reverse transcription and PCR steps to minimize PCR inhibition. Amplified cDNA was purified using AMPure XP beads (Beckman-Coulter) and quantitated using the Qubit Fluorometer and Qubit dsDNA HS Assay (Life Technologies). Purified dsDNA (2 ng) was used for NGS library generation using the Nextera XT kit (Illumina). Nextera XT libraries were purified using AMPure XP beads (Beckman Coulter) and sequenced on an Illumina HiSeq 2,500 in rapid mode using 150-bp paired-end sequencing. Samples were batched (12–16 samples per lane) and sequenced in parallel with negative controls (PBS) (one for each batch of samples) to monitor for potential reagent, laboratory, or cross-contamination.

### Bioinformatics analysis

Metagenomic next-generation sequencing (mNGS) data were analyzed for viral nucleic acids using SURPI + , a bioinformatics pipeline for pathogen detection and discovery from metagenomic data^14^, modified to incorporate enhanced filtering and classification algorithms^15^. The SNAP nucleotide aligner was run using an edit distance of 16 against the NCBI NT database containing the entirety of GenBank (March 2015)^16^. This enabled the detection of reads with ≥ 90% identity to reference sequences in the database.

Next, RAPSearch was used to screen for divergent viruses by translated nucleotide alignment to the NCBI nonredundant (NR) protein database (March 2015)^14^. In accordance with a prior clinical validation study^15^, the pre-established criterion for viral detection by SNAP or RAPSearch was the presence of reads mapping to at least 3 nonoverlapping regions of the viral genome^15^. In the current study, no highly divergent viruses were detected using RAPSearch.

For quantification of viral reads^8,14,17^, reads were normalized according to the number of preprocessed reads (adaptor-trimmed reads with exclusion of low-quality and low-complexity sequences) and expressed in reads per million (RPM). RPM values corresponding to presumptive viral laboratory, reagent, and/or cross-contaminants in the negative control samples were subtracted from clinical stool samples in the same sequencing batch, with negative RPM values after subtraction set to 0. To ensure accurate read counts, confirmation of reads corresponding to poliovirus was performed by manual BLASTn^18^ analysis using a stringent threshold e-value of 10^–20^. Reference-based assembly of poliovirus genomes was performed using Geneious 10.2.4 software (https://www.geneious.com/download/previous-versions/, accessed February 11th, 2020)^19^. Phylogenetic sequence analysis of recovered whole-genome poliovirus sequences in parallel with reference poliovirus Sabin 1–3 sequences (accession numbers AY184219, AY184220, AY184221) was also performed using Geneious 10.2.4 software^19^. Briefly, genome sequences were aligned using MAFFT^20^, followed by tree construction using PHYML^21^ at default settings.

Although the virome is predominantly populated with phages^22^, fewer prokaryotic than eukaryotic reads were identified on average in samples from this study. This is likely due to the use of a normalized RPM metric as a measure of relative (but not absolute) abundance, and the lack of precise species-specific taxonomic classification and sequence representation for phages relative to eukaryotic viruses in the GenBank reference database.

### Poliovirus antibody assay

Available serum samples from infants collected after the third OPV vaccination were assessed for neutralizing antibodies against human poliovirus Sabin 1–3 serotypes, as previously described^23,24^. Poliovirus neutralization was assessed by plaque-reduction neutralization testing (PRNT) using replication-competent poliovirus propagated in HeLa S3 cells over a period of 7 days^20^. Replication competent poliovirus was generated from infectious cDNA clones, as previously described^20^. HeLa S3 cells were seeded in 30 ml of 10% NCS DMEM/F12 48 h prior to infection with 7.5 ml of cDNA clones diluted with serum-free DMEM/F12. Virus was recovered after 3 infectious cycles (24 h) when cytopathic effect (CPE) is visible. Propagated virus was then titrated using TCID_50_ assay as previously described^20^. For poliovirus neutralization, 80 µL of serum was diluted with PBS and a series of twofold dilutions series was made (1/8 to 1/1,024 dilution) by adding 80 µL of DMEM/F12 into subsequent wells. Next, 80 μl of virus stocks diluted to 2000 TCID50/mL were added to each well. After a 90 min incubation, 100 μL of the virus and serum mix was transferred onto a plate seeded with 10,000 HeLa S3 cells/well and incubated for 7 days at 33 °C. After 7 days, plates were fixed with 2% formaldehyde and stained with 0.5% crystal violet. Neutralizing activity against each serotype was defined as 100% inhibition of infection (no plaques visualized after staining of the cell monolayer) at 1/8 dilution. The neutralizing antibody titers of the serum against human poliovirus serotype 1, 2 and 3 were determined as the highest dilution of the serum that produced 100% inhibition. Control sera for human poliovirus Sabin 1, 2, and 3 strains all demonstrated neutralizing antibodies at 1/8 dilution.

### Statistical analysis

Samples were considered positive for poliovirus if reads covered at least 3 gene regions of poliovirus genome^15^ and read counts were > 5X that of the negative control buffer sample (the “no-template” control or NTC). Based on manual inspection of the distribution of read frequencies, high poliovirus shedding was defined as ≥ 10 RPM. Categorical data were reported as counts and percentages and continuous data as medians with ranges, and significance tested by Fisher’s Exact and Mann–Whitney tests, respectively. Diversity metrics, including the Chao Richness Score, Shannon Diversity Index and Bray–Curtis dissimilarity between samples at the genus level were calculated using R package *vegan*2.5–3^25^. Principal coordinate analysis with Bray–Curtis dissimilarity was performed to visualize differences in community composition between groups based on degree of poliovirus shedding (based on the normalized RPM mapping to poliovirus in stool samples) and country of origin (Bangladesh or US), with significant differences assessed by permutation-based analysis of variance (PERMANOVA) using the Adonis function (default 1,000 permutations)^26^. Comparisons of virome abundance, richness and alpha diversity between groups were analyzed using either the Kruskal–Wallis rank sum test or a generalized estimating equations (GEE) model to account for the subject-structure of the longitudinal data and included age adjustment where applicable. All statistical tests were calculated as two-sided at the 0.05 significance level with virus family and genera associations adjusted for multiple comparisons using the Benjamini–Hochberg method for false discovery rate correction^27^. Analyses were performed using R version 3.3.3 software (RStudio version 1.1.383).

## Results

### Stool virome composition in Bangladeshi infants

Of 90 stool samples tested from 30 Bangladeshi infants, 87 were successfully sequenced (Table 1). Three samples failed amplification due to low nucleic acid concentration following extraction and were excluded from further analysis. Median ages of infants at the time of the first, second and third stool sampling were 9.9 (9.6–10.8), 12.0 (11.4–12.9) and 14.8 (14.4–15.8) weeks, respectively. Infants were sampled a median of 27 (24–28) days after administration of the first OPV vaccine and 9.5 (8–10) and 28.8 (27–30) days after the second OPV vaccination.

**Figure 2:**
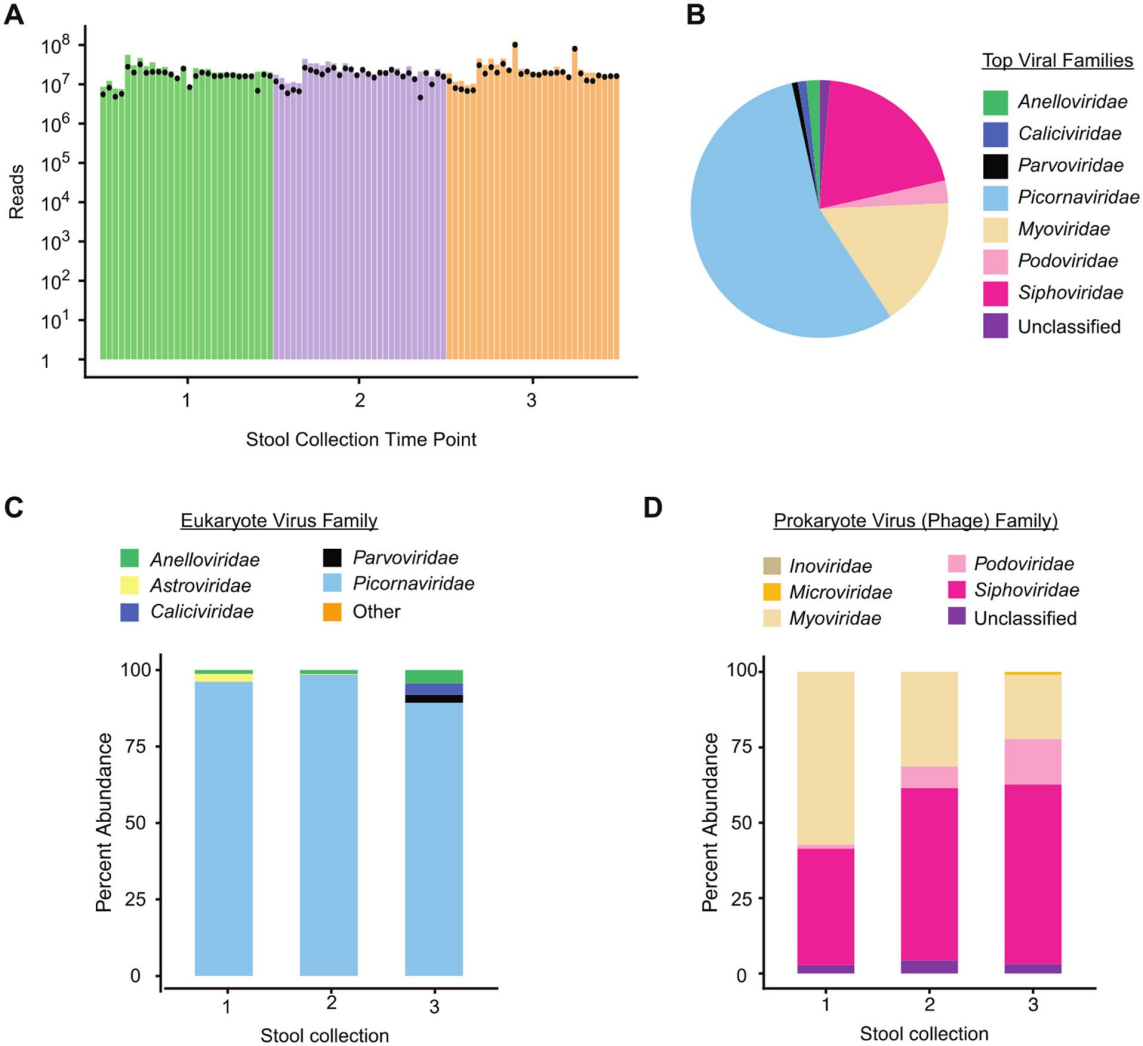
Stool Virome Composition in Bangladeshi Infants (A) Sequenced reads obtained per sample by stool collection time point. Dots reflect recovered number of reads after preprocessing raw data with trimming of adaptors and removing low-quality and low-complexity sequences. **(B)** Distribution of most abundant virus families reads in infant stool. **(C)** Percent abundance of eukaryotic vertebrate virus family by infant stool sample. **(D)** Percent abundance of phage family by infant stool sample.

**Figure 3:**
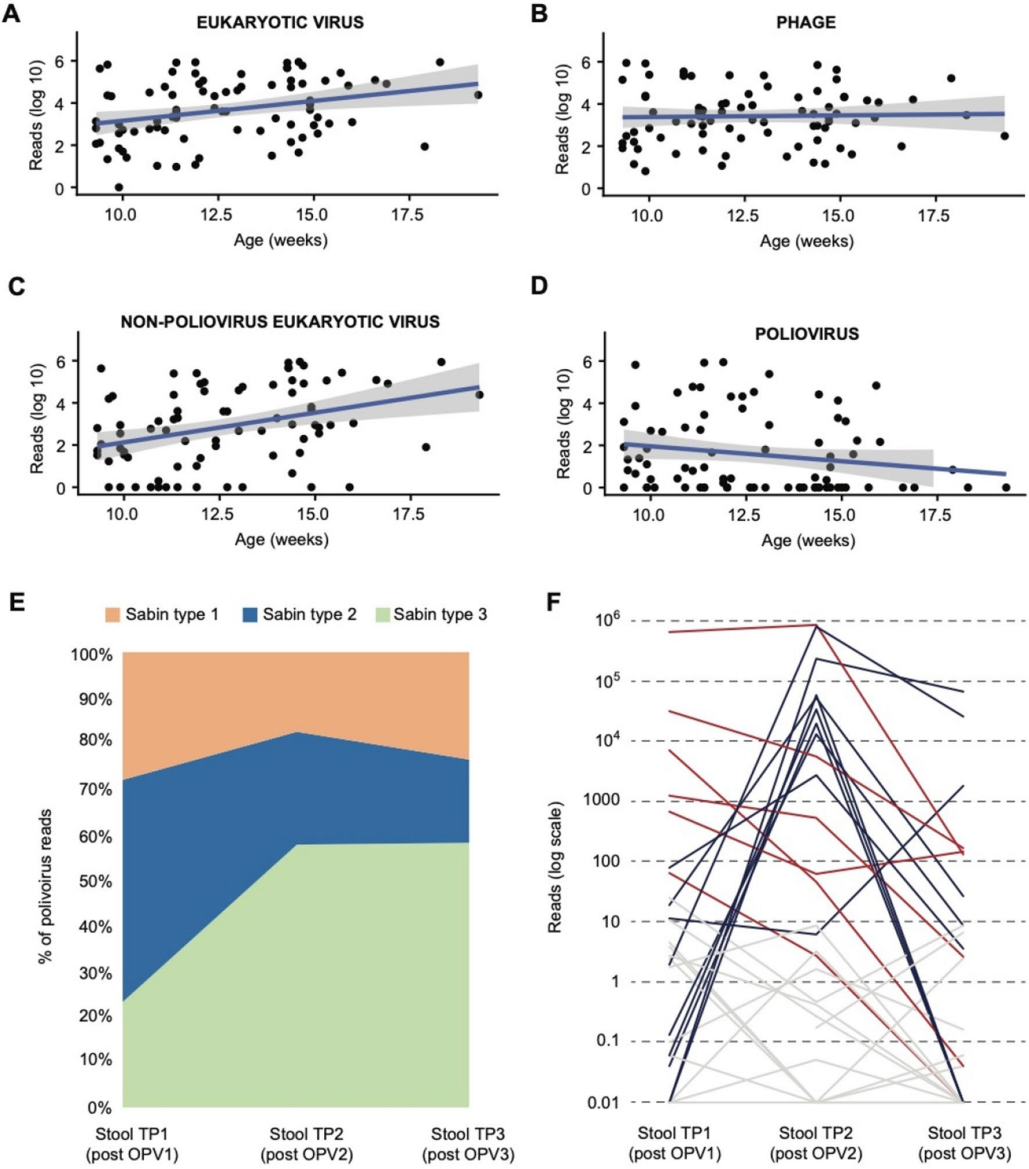
Viral abundance and poliovirus shedding. (A-D) Log-abundance of virus by infant age. **(A)** Eukaryote virus abundance. **(B)** Phage abundance **(C)** Non-poliovirus eukaryotic virus abundance **(D)** Poliovirus abundance; the regression line is plotted in blue, with the 95% confidence interval shown in gray. **(E)** Relative abundance of Sabin poliovirus strains at each time point following vaccination. The plot only includes data from subjects for whom samples at all 3 timepoints were available and ≥ 50 reads were detected in at least one timepoint. **(F)** Total poliovirus abundance at each time point. Colored lines denote infant stools with minimal viral shedding (gray), high shedding following second vaccine (blue), and high shedding following the first vaccine with gradual decline (red). Abbreviations: TP, time point, OPV, oral poliovirus vaccine.

Viral metagenomic sequencing analysis of the 87 stool samples yielded a total of 1.6 billion sequence reads, with a median of 17.6 million reads per sample (14.7–20.4 million; Fig. 2). By SURPI + analysis, the mean percentages of matched viral, bacterial and human reads across all samples were 12.26%, 23.36% and 10.71% of preprocessed reads, respectively (Supplementary Table S2). An average of 23.4% of reads did not map to any reference sequence in NCBI NT. Eukaryotic viruses of the vertebrate taxa (59.4%) and prokaryotic viruses (referred hereafter as phages) (40.6%) accounted for the majority of viral reads, with less than 0.01% of reads attributed to eukaryotic plant, invertebrate, fungi and protozoa viruses. Among eukaryotic viruses, the abundance as expressed by normalized reads per million (RPM) (heretofore referred to as “abundance”) increased with infant age (Pearson’s r = 0.30, p = 0.006) whereas phage abundance remained relatively static over time (Pearson’s r = 0.03, p = 0.88; Fig. 3A,B). The increase in eukaryotic viral abundance was driven by non-polioviruses (Pearson’s r = 0.358, p < 0.001) and was not due to the number of poliovirus reads, for which there was a non-significant opposite trend towards a decline in read counts with age (Pearson’s r = 0.18, p = 0.11) (Fig. 3C,D). Richness for eukaryotic viruses increased with age as well (p = 0.013), while alpha diversity for eukaryotic viruses and phages did not differ significantly over time (p = 0.41 and p = 0.70 respectively). No significant differences in total, eukaryotic or phage richness or abundance were observed among infants who had received weekly, biweekly, or no probiotics (Supplementary Table S3).

Among eukaryotic viruses, a total of 12 families, 33 genera and 179 species were identified. Picornaviruses accounted for the vast majority of reads (93.6%), followed by anelloviruses (2.7%), caliciviruses (1.8%) and parvoviruses (1.3%; Fig. 2). The majority of picornavirus reads mapped to the *Enterovirus* genus (62.1%) of which more than half (52.8%) aligned to polioviruses, followed by saliviruses (18.2%), parechoviruses (16.8%) and cosaviruses (2.8%). Notably, even after excluding poliovirus reads, enteroviruses remained the most abundant viruses identified. Cardioviruses and unclassified picornaviruses accounted for < 1% of picornavirus reads. Of the detected caliciviruses, norovirus represented 69.1% and sapovirus 30.9%. Bocaviruses comprised 100% of parvoviruses found in infant stool.

All infants had picornaviruses in at least one stool sample, with 90% of infants shedding poliovirus during at least one of the sampled timepoints. Anelloviruses and circoviruses were found in 96.7% and 76.6% of infants, respectively, followed by caliciviruses (33.3%), papillomaviruses (23.3%) and astroviruses (23.3%). Other viruses, including herpesviruses, (13.3%), adenoviruses (6.7%), hepatitis B virus (3.3%) and MW polyomavirus (3.3%), were less commonly detected in infant stool.

Many of the viruses identified in stool from Bangladeshi infants, including norovirus, sapovirus, mamastrovirus, salivirus, rotavirus, bocavirus, adenovirus, cosavirus, parechovirus, or cytomegalovirus, were known or suspected pathogens associated with acute diarrheal or respiratory infection (Supplementary Table S4). The majority (86.7%) of infants shed at least one of these viruses on at least one occasion. Nearly two-thirds of infants (63.3%) had norovirus, sapovirus, astrovirus, salivirus and/or rotavirus detected in at least one sample, whereas nearly half (43.3%) had bocavirus, adenovirus, cosavirus, parechovirus and/or cytomegalovirus detected. MW polyomavirus was identified in one infant and hepatitis B virus in another. Across the sampling period, infants shed on average 1.5 (range 0–6) potential viral pathogens in their stool samples. At the time of stool collection, infants were symptomatic (defined as one or more of fever (T > 38 °C), cough, congestion, vomiting, or watery diarrhea based on a documented clinical assessment by a nurse or physician) for 34 of 87 time points (39.1%) (Supplementary Table S5). Symptomatic infants had a putative viral pathogen detected in stool approximately half of the time (18/34, 52.9%) (Supplementary Table S5). Conversely, the presence of a viral pathogen in stool was only occasionally associated with symptoms (18/50, 36%), with most identified viruses found in asymptomatic infants (32/50, 64%). Overall, there was no apparent correlation between symptoms and detection of pathogenic viruses in stool (p = 0.51, Fisher’s Exact Test). During the study period, only one infant was hospitalized; this child presented with fever and cough and had a high number of parechovirus reads (269,383 RPM, Supplementary Table S5, P167) detected in stool at the time of acute illness.

Among phages, 5 families, 30 genera and 541 species were identified. Phages in infant stool were primarily caudoviruses belonging to the siphovirus (49.3%), myovirus (40.2%) and podovirus (6.9%) families. Phage species most predominantly identified were unclassified siphoviruses*,* followed by phages from bacterial species largely comprising gastrointestinal flora.

### Stool poliovirus shedding varies among Bangladeshi infants

Polioviruses accounted for 31.7% of eukaryotic virus reads; 63.2% (55/87) of samples were positive for poliovirus, of which 65.4% (36/55) of samples were classified as having high poliovirus reads (> 10 RPM). 70.4% of vaccinated infants shed poliovirus in stool 30 days from the first vaccine (time point 1); these infants predominantly shed Sabin type 2 and 3 polioviruses with all simultaneously shedding multiple Sabin types at roughly equal overall proportion (Table 2). At the second stool sampling (time point 2, median 10 days after second OPV vaccination), 74.1% of infants predominantly shed Sabin type 3 poliovirus (60%), with shedding of multiple Sabin types seen in 75%. Poliovirus shedding was reduced to 48% of infants by the third stool sample (time point 3, median 29 days after second vaccination). Overall, the relative proportion of poliovirus reads comprising Sabin poliovirus types 1, 2, and 3 were roughly equal at time point 1, with the relative proportion of Sabin type 3 poliovirus increasing with a concomitant decrease in Sabin type 2 poliovirus by time points 2 and 3 (Fig. 3E).

**Table 2:** Poliovirus shedding characteristics among stool samples.

Stool sample	Median days (range) after vaccine	% shedders	% of the most common poliovirus strain detected in shedders^c^		
			Sabin 1	Sabin 2	Sabin 3
First sample (n = 29)	27 (24–48)^a^	70.4%	15.8%	47.4%	36.8%
Second sample (n = 30)	9.5 (8–10)^b^	74.1%	15%	25%	60%
Third sample (n = 28)	28.8 (27–30)^b^	48.3%	15.4%	15.4%	69.2%

^a^After first poliovirus vaccine.

^b^After second poliovirus vaccine.

^c^Defined as the number of subjects shedding Sabin 1, 2, or 3 as the dominant poliovirus strain (strains with the greatest proportion of mapped reads) divided by the total number of subjects with poliovirus detected in stool.

The kinetics of poliovirus shedding differed among infants with three dominant patterns observed: (1) minimal or no shedding of vaccine at any time point (n = 11), (2) high shedding soon after second vaccine but not after the first (n = 13) and high shedding after first vaccine that gradually declined after second vaccine (n = 6) (Fig. 3F).

**Figure 4:**
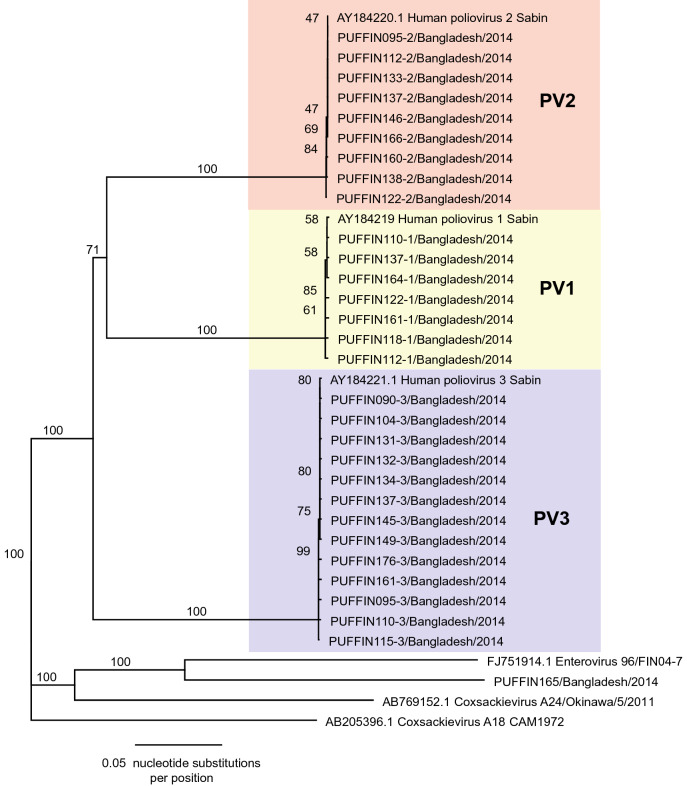
Phylogenetic analysis of whole genome poliovirus sequences. Comparison of 29 whole genome sequences to vaccine reference strains revealed viruses with percent pairwise identity of 100% for Sabin 1 and 3 and 100% identity for Sabin 2 viruses (differences only due to slight variations in total coverage) and no significant vaccine-derived poliovirus variants.

Viral metagenomic sequencing of infant stool allowed for the assembly of 29 whole genome consensus poliovirus sequences. Comparison of whole genome sequences to vaccine reference strains revealed viruses that were identical (Fig. 4). Thus, no significant vaccine-derived poliovirus variants were observed in this cohort.

According to Bray–Curtis dissimilarity scores, the eukaryotic virome composition of infant stools containing poliovirus differed significantly from those that did not (p = 0.007), even after exclusion of poliovirus reads, whereas the phage virome did not differ between groups (p = 0.50). In particular, abundance (3.68 log_10_ vs. 2.25 log_10_, p = 0.002) and richness (p = 0.016) of non-poliovirus eukaryotic viruses, particularly those associated with acute respiratory or gastrointestinal illness (2.78log_10_ vs. 0.83log_10_, p = 0.002), was inversely associated with poliovirus shedding (Supplementary Table S6). Phage abundance (3.47log_10_ vs, 3.37log_10_, p = 0.50), richness (p = 0.06) and alpha diversity (p = 0.07) also did not significantly differ between groups (Supplementary Table S6). No specific virus genus or family was significantly associated with shedding of poliovirus after false discovery rate correction (Supplementary Tables S7 and S8). No associations between infant sex, maternal education, economic status, or breastfeeding status (exclusive breastfeeding or partial breastfeeding with supplementation) with poliovirus or other non-poliovirus eukaryotic viruses were observed (Supplementary Table S9).

### Low neutralizing antibody response to poliovirus in Bangladeshi infants

Serum available from 14 infants, collected a median of 31 days (range: 14–34) after administration of third OPV vaccine, was tested for neutralizing antibodies against all three Sabin poliovirus types. The median infant age at time of collection was 19.6 (range 16.3–24.3) weeks old. All 14 infants had detectable neutralizing antibodies to at least one Sabin type, although only four infants (28.6%) developed antibodies to all three types (Supplementary Table S10). Infants who had neutralizing antibodies to only one Sabin type had significantly lower stool poliovirus reads during the sampled time points compared to those who developed neutralizing antibodies to all three types (0.36log_10_ vs. 3.72log_10_, p = 0.02, (Supplementary Table S11). Evaluation of virus genera, abundance, richness and diversity did not reveal significant differences among the infants among the varied serologic outcomes (Supplementary Table S12).

### Stool virome composition is distinct in Bangladeshi compared to US infants

**Figure 5:**
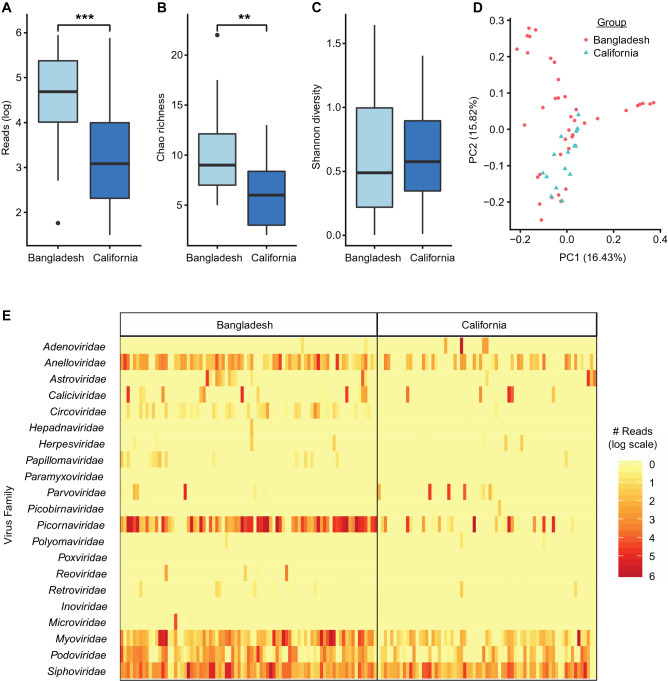
Comparison of stool virome in Bangladeshi and California infants. (A) After exclusion of poliovirus reads, virome abundance and (**B)** richness (Chao) were significantly higher in Bangladeshi infants compared to California (USA) infants (p < 0.001). **(C)** Alpha diversity (Shannon) was not significantly different between groups (p = 0.27). **(D)** Principal coordinates analysis of Bray–Curtis dissimilarity shows co-clustering of Bangladeshi and California infants (p = 0.002 by PERMANOVA analysis). **(E)** Heat map showing distribution of virus families at each geographic site.

A total of 40 stool samples from the Bangladeshi study were age-matched (12–16 weeks of age) to stool samples collected from 16 California infants. Comparison of the stool samples from Bangladeshi versus California infants demonstrated marked differences in virome composition (p = 0.002 by PERMANOVA analysis; Fig. 5, Supplementary Table S13). Total virome abundance (4.69log_10_ vs. 3.08log_10_, p = 0.004) and richness (p = 0.01) were significantly higher in Bangladeshi infants, in large part due to increased abundance (4.22log_10_ vs. 1.51log_10_, p < 0.001) and richness (p = 0.003) of the eukaryotic virome. Since OPV is no longer given to US children, poliovirus shedding was exclusively seen in Bangladeshi infants. After removing polioviruses from the analysis, the abundance (3.43log_10_ vs. 1.51log_10_, p = 0.003) and richness (p = 0.005) of the non-poliovirus eukaryotic virome remained significantly higher in Bangladeshi infants. In contrast, phage abundance (3.44log_10_ vs. 3.08log_10_, p = 0.14), richness (p = 0.10) and alpha diversity (p = 0.27) did not differ significantly between Bangladeshi and US infants.

## Discussion

In this study, we characterized the gut virome of Bangladeshi infants and evaluated its association with poliovirus shedding. Consistent with prevailing hypotheses^2,29,30^, we found that shedding of enteric viral pathogens is associated with decreased poliovirus shedding. However, unlike prior studies^2,23^, the observed association was not confined to nonpolio enteroviruses alone^2^. Bangladeshi infants with decreased or no shedding of poliovirus following vaccination exhibited a higher abundance of eukaryotic viruses overall, particularly viruses that are known causes of gastrointestinal or respiratory illness, than infants with higher rates of shedding.

Here we also found a direct correlation between the degree of poliovirus shedding and the development of neutralizing antibody responses, as previously observed^31^. Notably, only 28.6% (4 of 14) of infants generated neutralizing antibodies to all three Sabin types in the current study, lower than the 60% (33 of 55) seroconversion rate previously reported from India^32^ and nearly 100% seroconversion rate in Western populations^33^. Given the low numbers, the differences in observed seroconversion rate between Indian infants from the 1970’s^32^ or Bangladeshi infants in the current study are not statistically significant at a p-value threshold of 0.05 (p = 0.0693 by Fisher’s Exact Test). These differences are very likely multi-factorial^24^ and may reflect, among other possibilities, variability in environmental exposures and/or probiotic administration in our study cohort. Taken together, our results suggest that eukaryotic virome abundance may contribute to the diminished OPV seroconversion rates observed in low socioeconomic settings, such as Bangladesh. However, in the current study, we did not observe a significant association between virome metrics and OPV seroconversion, perhaps due to the low number of samples (n = 14) available for serological analysis.

Compared to similarly aged infants from the United States, the gut virome of Bangladeshi infants relative to that of US children was strikingly more abundant and richer in non-poliovirus eukaryotic viruses. This variation is likely due to a combination of factors, including differences in culture, urbanization, socioeconomic status and geography^34^. Although viral pathogens were commonly shed, only ~ 36% were detected in symptomatic individuals, underscoring the significant contribution of asymptomatic infection or colonization to the virome. In addition to viruses known to cause respiratory or gastrointestinal illness, nearly a quarter of Bangladeshi infants shed human papillomavirus and one infant had hepatitis B virus identified in stool, suggesting perinatal acquisition. In contrast, the phage virome in Bangladeshi and US infants were comparable in abundance, richness and diversity. Caudoviruses comprised the majority of the phage virome, consistent with prior reports of bacteriophages in breast milk and infant stool and a shift in from *Caudovirales*-dominated to *Microviridae*-dominated communities over the first 2 years of life^5,35^.

This study used a viral metagenomic next-generation sequencing approach to evaluate gut virome associations with poliovirus shedding and oral vaccine response. Current literature evaluating potential interference of oral poliovirus vaccine responses by enteric viruses has relied on targeted molecular or culture-based detection methods, providing only data on a limited number of individual viruses^2,29,30^. Our data suggest that the abundance and richness of the virome in its entirety may play a more important role in poliovirus vaccine responses than infection by any specific viral pathogen. Metagenomic virome analysis of poliovirus sequences also allowed us to document shedding of multiple poliovirus Sabin serotypes, recover 29 whole poliovirus genome sequences and determine that no vaccine-derived poliovirus variants or vaccine revertants were evident in this cohort.

This study had some limitations. First, a weakness of the study is that no samples were available for analysis before the administration of OPV, so a baseline virome could not be determined. Thus, it is possible that poliovirus replication may also alter the composition of the virome, in addition to the baseline virome potentially affecting the rates of poliovirus shedding. Second, the number of samples and time points remains limited. Testing of infants over a longer period of time and with additional time points would have enhanced power for multiple comparisons and enabled more detailed evaluation of virome dynamics. Associations between maternal, environmental and diet exposures^34,36,37^ would also more likely be uncovered in a larger study. In addition, variability in sampling times in the setting of an evolving virome may have decreased the power of the study to detect associations between the virome, poliovirus shedding, and OPV seroconversion. Third, potential bias by varying exposure to probiotics and selection of only an available subset (30 of 160) of Bangladeshi infants may have affected the results of the study. Of note, probiotic organisms were cultured only rarely from infant stool, even soon after administration (KJ and JP, personal communication), and it is more likely that the use of probiotics would disproportionately affect the phage rather than eukaryotic virome. Fourth, phage identification was determined using nucleotide similarity to available, taxonomically classified reference sequences in the GenBank database. This likely underestimated the true phage population, as the phage sequence database is incomplete and the vast majority of phages remain unclassified^38^. Finally, serum samples were also available for only 14 (47%) infants at a single time point, limiting interpretation of our study results; more work is needed to establish the impact of the virome on poliovirus vaccine serological responsiveness over time.

In summary, this study characterizes the gut virome composition in Bangladeshi infants following poliovirus vaccination. Metagenomic virus sequencing revealed dynamic exposures to eukaryotic vertebrate viruses, which were found to be inversely associated with poliovirus shedding. This finding lends support to the premise that the gut virome composition and infection from viruses other than poliovirus may contribute to oral vaccine responsiveness in infants.

## Supplementary information

Supplementary Tables

Supplementary Table S5

## Data Availability

Reads with human sequences removed by Bowtie2^28^ high-sensitivity local alignment to the human genome (GRChg38/hg38 build) have been deposited in the NCBI Sequence Read Archive (SRA) (PRJNA644725). Poliovirus genome sequences have been deposited in NCBI GenBank (MT957178-MT957207).
