# Supplementary material for: Metagenomic sequencing of stool samples in Bangladeshi infants: virome association with poliovirus shedding after oral poliovirus vaccination
Source: Sci Rep. 2020 Sep 21;10:15392. doi: 10.1038/s41598-020-71791-4 (PMC7506025; doi:10.1038/s41598-020-71791-4)
Supplement: Supplementary file 1 — Supplementary Tables [file 41598_2020_71791_MOESM1_ESM.docx]

**SUPPLEMENTARY MATERIAL**

**Metagenomic Sequencing of Stool Samples in Bangladeshi Infants: Virome Association with Poliovirus Shedding After Oral Poliovirus Vaccination**

Susanna K. Tan^1&^, Andrea Granados^2,3&^, Jerome Bouquet^2,3^, Yana Emmy Hoy-Schulz^1^, Lauri Green^2,3^, Scot Federman^2,3^, Doug Stryke^2,3^, Thomas D Haggerty^1^, Catherine Ley^1^, Ming-Te Yeh^4^, Kaniz Jannat^5^, Yvonne A. Maldonado^6^, Raul Andino^4^, Julie Parsonnet^1,8&^, Charles Y. Chiu^2,3,9&*^

^1^Department of Medicine, Division of Infectious Diseases, Stanford University School of Medicine, Stanford, California, USA

^2^Department of Laboratory Medicine, University of California, San Francisco, California, USA

^3^UCSF-Abbott Viral Diagnostics and Discovery Center, San Francisco, California, USA

^4^Department of Microbiology and Immunology, University of California, San Francisco, California, USA

^4^Department of Microbiology and Immunology, University of California, San Francisco, California, USA

^5^Environmental Intervention Unit, Infectious Disease Division, International Centre for Diarrheal Disease Research, Dhaka, Bangladesh

^6^Department of Pediatrics, Division of Infectious Diseases, Stanford University School of Medicine, Stanford, California, USA

^7^Department of Epidemiology and Population Health, Stanford University School of Medicine, Stanford, California, USA

^8^Department of Epidemiology and Population Health, Stanford University School of Medicine, Stanford, California, USA

^9^Department of Medicine, Division of Infectious Diseases, University of California, San Francisco, California, USA

^&^These authors contributed equally.

*corresponding author

| **Supplementary Table S1. Immunization program for Bangladeshi Infants** | |  |
| --- | --- | --- |
|  |  |  |
| **Vaccine** | **Age at Administration** |  |
| BCG | At birth |  |
| pentavalent^a^ | 6 weeks, 10 weeks, 14 weeks |  |
| OPV | 6 weeks, 10 weeks, 14 weeks, 38 weeks |  |
| PCV | 6 weeks, 10 weeks, 14 weeks |  |
| IPV^b^ | 14 weeks |  |
| MR | 38 weeks |  |
| Measles | 15 months |  |
| Abbreviations: BCG; Bacillus Calmette-Guerin, OPV; oral polio vaccine, PCV; pneumococcal conjugate vaccine, IPV; injectable polio vaccine, MR; measles-rubella vaccine | | |
| ^a^ pentavalent vaccine includes diphtheria, tetanus, pertussis, Haemophilus influenzae type B, and hepatitis B virus | | |
| ^b^ used in conjunction in OPV | | |

**Supplementary Table S2. Number of reads sequenced from stool of Bangladeshi and US infants.**

|  | **PUFFIN1 (n=14)** | **PUFFIN2 (n=14)** | **PUFFIN3 (n=15)** | **PUFFIN4 (n=16)** | **PUFFIN5 (n=15)** | **PUFFIN6 (n=15)** | **PUFFIN7 (n=14)** | **PUFFIN8 (n=3)** | **PUFFIN9 (n=16)** | **PUFFIN10 (n=16)** | **PUFFIN11 (n=16)** | **PUFFIN12 (n=16)** | **Average ± SD** |
| --- | --- | --- | --- | --- | --- | --- | --- | --- | --- | --- | --- | --- | --- |
| **Raw Data** | 300,502,038 | 306,764,648 | 408,385,888 | 30,313,918 | 97,693,544 | 309,293,924 | 343,472,672 | 345,666,588 | 338,877,476 | 332,304,082 | 304,882,158 | 312,819,964 | 285,914,742 ±1.09E+8 |
| **Preprocessed** | 248,388,307 | 248,256,299 | 303,273,921 | 19,281,782 | 61,361,860 | 223,644,729 | 198,350,352 | 299,591,514 | 284,117,969 | 189,585,573 | 187,546,743 | 166,349,511 | 202,479,047 ± 8.85E+7 |
| **Human Matched** | 19,742,182 | 13,768,527 | 21,603,496 | 1,556,041 | 1,460,576 | 9,922,023 | 12,464,105 | 122,826,958 | 6,495,263 | 32,143,333 | 6,415,484 | 11,713,883 | 21,675,989 ± 3.33E+7 |
| **NT Matched** | 114,686,254 | 117,412,313 | 134,248,012 | 6,228,281 | 17,159,597 | 69,609,074 | 86,912,205 | 92,903,749 | 95,066,849 | 68,563,242 | 39,466,453 | 47,011,388 | 74,105,618 ± 4.04E+7 |
| **NT Virus Matched** | 44,293,507 | 63,155,049 | 60,386,774 | 2,939,811 | 13,886,171 | 38,534,300 | 43,754,501 | 454,798 | 4,502,526 | 20,549,013 | 2,359,221 | 2,957,522 | 24,814,433 ± 2.39E+7 |
| **NT Bacteria Matched** | 66,699,908 | 53,690,992 | 71,410,993 | 2,922,736 | 3,171,764 | 30,509,719 | 42,520,396 | 89,761,353 | 86,762,463 | 42,395,098 | 35,976,637 | 41,779,761 | 47,300,152 ± 2.82E+7 |
| **Unmatched** | 66,699,908 | 53,690,992 | 71,410,993 | 2,922,736 | 3,171,764 | 30,509,719 | 42,520,396 | 89,761,353 | 86,762,463 | 42,395,098 | 35,976,637 | 41,779,761 | 47,300,152 ± 2.92E+7 |
| **% Unmatched** | 26.9% | 21.6% | 23.5% | 15.2% | 5.2% | 13.6% | 21.4% | 30.0% | 30.5% | 22.4% | 19.2% | 25.1% | 23.4% |

Abbreviations: NT, NCBI nucleotide (“NT”) database; PUFFINx, sequencing runs 1-12 from the 46 infants (30 Bangladeshi, 16 California) in the PUFFIN study.

**Supplementary Table S3. Viral richness and abundance according to probiotic use in study infants**

| **Virome Metric** | **weekly probiotics (n=13)**  **median (SD)** | **semiweekly probiotics (n=12)**  **median (SD)** | **no probiotics (control) (n=5)**  **median (SD)** | **p-value^a^** |
| --- | --- | --- | --- | --- |
| Chao richness – total | 10 (±1.48) | 9.73 (±1.03) | 9.72 (±1.04) | 0.81 |
| Chao richness – eukaryotic virus | 4.71(±1.55) | 4.61(±1.44) | 5.03 (±1.34) | 0.705 |
| Chao richness – phage | 5.21 (±1.48) | 4.97(±1.24) | 4.6 (±1.36) | 0.716 |
|  |  |  |  |  |
| Shannon diversity – total | 0.63(±0.42) | 0.55 (±0.41) | 0.78 (±0.38) | 0.339 |
| Shannon diversity – eukaryotic virus | 0.37(±0.45) | 0.39 (±0.45) | 0.47(±0.51) | 0.633 |
| Shannon diversity – phage | 0.71(0.39) | 0.46(±0.38) | 0.58(±0.36) | 0.073 |

^a^ calculated using the Kruskal-Wallis rank sum test

**Supplementary Table S4. Detection of putative viral pathogens in stool from Bangladeshi infants**

|  | **Infant #** | | | | | | | | | | | | | | | | | | | | | | | | | | | | | |  |
| --- | --- | --- | --- | --- | --- | --- | --- | --- | --- | --- | --- | --- | --- | --- | --- | --- | --- | --- | --- | --- | --- | --- | --- | --- | --- | --- | --- | --- | --- | --- | --- |
|  | **1** | **2** | **3** | **4** | **5** | **6** | **7** | **8** | **9** | **10** | **11** | **12** | **13** | **14** | **15** | **16** | **17** | **18** | **19** | **20** | **21** | **22** | **23** | **24** | **25** | **26** | **27** | **28** | **29** | **30** | **Sum (infants)** |
| **Adenovirus** | 0 | 0 | 0 | 0 | 0 | 0 | 0 | 0 | 0 | 0 | 0 | 0 | 0 | 0 | 0 | 0 | 0 | 0 | 0 | 0 | 0 | 0 | 1 | 0 | 0 | 0 | 0 | 0 | 1 | 0 | 2 |
| **Cytomegalovirus** | 0 | 0 | 1 | 0 | 1 | 0 | 0 | 0 | 0 | 0 | 0 | 0 | 0 | 0 | 0 | 1 | 0 | 0 | 0 | 0 | 0 | 0 | 0 | 0 | 0 | 1 | 0 | 0 | 0 | 0 | 4 |
| **Bocavirus** | 0 | 0 | 0 | 0 | 0 | 0 | 0 | 1 | 0 | 0 | 0 | 0 | 0 | 0 | 0 | 0 | 0 | 1 | 0 | 0 | 0 | 0 | 0 | 0 | 0 | 0 | 0 | 0 | 0 | 0 | 2 |
| **Cosavirus** | 0 | 0 | 0 | 0 | 0 | 0 | 0 | 0 | 0 | 0 | 0 | 0 | 0 | 0 | 0 | 0 | 0 | 0 | 0 | 0 | 0 | 1 | 0 | 1 | 0 | 0 | 0 | 0 | 0 | 0 | 2 |
| **Parechovirus** | 0 | 0 | 1 | 0 | 0 | 0 | 0 | 0 | 0 | 0 | 0 | 0 | 0 | 1 | 1 | 1 | 0 | 0 | 0 | 0 | 0 | 1 | 0 | 0 | 0 | 0 | 1 | 1 | 1 | 1 | 9 |
| **Astrovirus** | 0 | 0 | 0 | 0 | 0 | 0 | 0 | 0 | 0 | 0 | 1 | 1 | 1 | 1 | 0 | 1 | 0 | 1 | 0 | 0 | 0 | 0 | 0 | 0 | 0 | 0 | 0 | 0 | 1 | 0 | 7 |
| **Norovirus** | 0 | 1 | 0 | 1 | 0 | 0 | 0 | 0 | 0 | 1 | 0 | 0 | 0 | 1 | 0 | 0 | 0 | 1 | 0 | 0 | 0 | 0 | 0 | 0 | 0 | 0 | 1 | 0 | 1 | 0 | 7 |
| **Sapporo virus** | 0 | 1 | 1 | 0 | 0 | 1 | 0 | 0 | 0 | 1 | 0 | 0 | 0 | 0 | 0 | 0 | 0 | 0 | 0 | 0 | 0 | 0 | 0 | 0 | 1 | 0 | 0 | 0 | 1 | 0 | 6 |
| **Salivirus** | 0 | 0 | 0 | 0 | 0 | 0 | 1 | 0 | 0 | 0 | 0 | 0 | 0 | 1 | 1 | 1 | 1 | 1 | 0 | 0 | 0 | 0 | 0 | 1 | 0 | 1 | 0 | 1 | 0 | 1 | 10 |
| **Rotavirus** | 0 | 0 | 0 | 0 | 0 | 1 | 0 | 0 | 0 | 0 | 0 | 0 | 0 | 0 | 0 | 1 | 0 | 0 | 0 | 0 | 1 | 0 | 0 | 0 | 0 | 0 | 0 | 0 | 0 | 0 | 3 |
| **Sum (viruses)** | 0 | 2 | 3 | 1 | 1 | 2 | 1 | 1 | 0 | 2 | 1 | 1 | 1 | 4 | 2 | 5 | 1 | 4 | 0 | 0 | 1 | 2 | 1 | 2 | 1 | 2 | 2 | 2 | 5 | 2 |  |

**Supplementary Table S5. List of symptoms and putative viral pathogens in stool for each Bangladeshi infant in the study.**

“Supplementary Table_S5.xlsx”

**Supplementary Table S6. Virome associations with poliovirus shedding status**

|  | **Polio Shedding Status**^b^ | | | **Polio Shedding Status**^b^ | | |
| --- | --- | --- | --- | --- | --- | --- |
| **Virome Metric** | No (n=33)  median (SD) | Yes (=54)  median (SD) | p-value^c^ | Low (N=17)  median (SD) | High (n=37)  median (SD) | p-value^c^ |
| Total Log Abundance | 4.61(±1.34) | 4.53 (±1.27) | 0.517 | 4.44(±1.37) | 4.56(±1.09) | 0.190 |
| Prokaryotic Virus Log Abundance | 3.47(±1.80) | 3.37(±1.55) | 0.500 | 3.11(±1.66) | 3.49(±1.54) | 0.330 |
| Eukaryotic Virus Log Abundance | 3.68(±1.78) | 2.25(±1.37) | **0.002** | 2.73(±1.68) | 2.20(±1.16) | 0.096 |
|  |  |  |  |  |  |  |
| Log Abundance GI Virus+Resp Virus^a^ | 2.78(±1.99) | 0.83(±0.66) | **0.002** | 0.00(±1.87) | 0.00(±1.22) | 0.400 |
|  |  |  |  |  |  |  |
| Chao Richness (Total) | 11(±4.76) | 9(±3.48) | 0.050 | 9.5(±3.25) | 8.5(±4.16) | 0.820 |
| Chao Richness (Prokaryotic Virus) | 5(±2.38) | 4(±2.74) | 0.055 | 5(±2.38) | 4(±2.26) | 0.730 |
| Chao Richness (Non-Polio Eukaryotic Virus) | 5(±1.88) | 4(±2.49) | **0.016** | 5(±2.47) | 4(±2.34) | 0.210 |
|  |  |  |  |  |  |  |
| Shannon Diversity (Total) | 0.64(±0.45) | 0.45(±0.22) | 0.201 | 0.30(±0.22) | 0.49(±0.27) | 0.580 |
| Shannon Diversity (Prokaryotic Virus) | 0.67(±0.45) | 0.5(±0.29) | 0.069 | 0.57(±0.39) | 0.47(±0.30) | 0.430 |
| Shannon Diversity (Non-Polio Eukaryotic Virus) | 0.36(±0.42) | 0.48(±0.33) | 0.533 | 0.36(±0.41) | 0.50(±0.41) | 0.190 |

^a^ defined as detection of adenovirus, astrovirus, bocavirus, cosavirus, cytomegalovirus, norovirus, parechovirus, sapoirus, salivirus and/or rotavirus in stool sample (GIVirus+RespVirus)

^b^ definitions of poliovirus shedding status: No, <3 non-overlapping poliovirus reads; Yes, ≥3 non-overlapping poliovirus; Low, ≥3 non-overlapping poliovirus reads but <10 poliovirus RPM; High, ≥10 poliovirus RPM

^c^ calculated using the Kruskal-Wallis rank sum test

**Supplementary Table S7. Virus family associations with poliovirus shedding status.**

| **Virus Family** | **p-value*** |
| --- | --- |
| *Adenoviridae* | 0.711 |
| *Anelloviridae* | 0.711 |
| *Astroviridae* | 0.711 |
| *Caliciviridae* | 0.711 |
| *Circoviridae* | 0.738 |
| *Hepadnaviridae* | 0.80 |
| *Papillomaviridae* | 0.74 |
| *Parvoviridae* | 0.711 |
| *Picornaviridae* | 0.711 |
| *Reoviridae* | 0.711 |
| *Retroviridae* | 0.9 |
| *Inoviridae* | 0.711 |
| *Microviridae* | 0.727 |
| *Myoviridae* | 0.9 |
| *Podoviridae* | 0.727 |
| *Siphoviridae* | 0.711 |

* calculated using Fisher’s Exact Test; p-value adjusted for multiple comparisons using the Benjamini-Hochberg method^1^

**Supplementary Table S8. Virus genus associations with poliovirus shedding status.**

| **Virus Genus** | **p-value*** |
| --- | --- |
| Alphatorquevirus | 0.917 |
| Betatorquevirus | 0.500 |
| Gammatorquevirus | 0.138 |
| Unclassified_Anelloviridae | 0.917 |
| Mamastrovirus | 0.500 |
| Unclassified_Astroviridae | 0.354 |
| Norovirus | 0.338 |
| Sapovirus | 0.917 |
| Unclassified_Caliciviridae | 0.183 |
| Circovirus | 0.809 |
| Unclassified_Circoviridae | 0.917 |
| Orthohepadnavirus | 0.917 |
| Cytomegalovirus | 0.635 |
| Betapapillomavirus | 0.917 |
| Dependoparvovirus | 0.275 |
| Erythroparvovirus | 0.809 |
| Unclassified_Parvovirida | 0.917 |
| Cardiovirus | 0.500 |
| Cosavirus | 0.809 |
| Erbovirus | 0.500 |
| Parechovirus | 0.275 |
| Salivirus | 0.917 |
| Polyomavirus | 0.635 |
| Aquareoviru | 0.917 |
| Rotavirus | 0.917 |
| Gammaretrovirus | 0.809 |
| Unclassified_Retroviridae | 0.917 |
| Inovirus | 0.500 |
| Microvirus | 0.635 |
| Bcepmulikevirus | 0.917 |
| Felixounalikevirus | 0.917 |
| Hpunalikevirus | 0.917 |
| Mulikevirus | 0.917 |
| P2likevirus | 0.917 |
| PhiCD119likevirus | 0.500 |
| Punalikevirus | 0.275 |
| T4likevirus | 0.110 |
| Twortlikevirus | 0.275 |
| Unclassified_Myoviridae | 1.000 |
| Viunalikevirus | 0.367 |
| Ahjdlikevirus | 0.809 |
| Epsilon15likevirus | 0.917 |
| N4likevirus | 0.147 |
| P22likevirus | 0.183 |
| Phi29likevirus | 0.809 |
| Sp6likevirus | 0.917 |
| T7likevirus | 0.917 |
| Unclassified_Podoviridae | 0.809 |
| C2likevirus | 0.500 |
| Lambdalikevirus | 0.917 |
| N15likevirus | 0.275 |
| T5likevirus | 0.183 |
| Tunalikevirus | 0.809 |
| Unclassified_Siphoviridae | 0.635 |
| Phikmvlikevirus | 0.275 |

* calculated using Fisher’s Exact Test; p-value adjusted for multiple comparisons using the Benjamini-Hochberg method^1^

**Supplementary Table S9. Virome associations with demographic characteristics.**

|  | **Sex** | |  | **Maternal education** | |  | **Economic class** | |  | **Breastfed status** | |  |
| --- | --- | --- | --- | --- | --- | --- | --- | --- | --- | --- | --- | --- |
| **Virome Metric** | Male (n=16)  median (SD) | Female (n=14)  median (SD) | p-value^a^ | <6 years (n=9)  median (SD) | ≥ 6 years (n=18)  median (SD) | p-value^a^ | <$100/month  (n=23)  median (SD) | >$100/month (n=7)  median (SD) | p-value^a^ | Partial (n=20)  median (SD) | Exclusive (n=10)  median (SD) | p-value^a^ |
| Log Abundance (Total) | 4.61 (±1.36) | 4.54 (±0.91) | 0.300 | 4.73 (±1.26) | 4.5 (±0.93) | 0.970 | 4.51 (±1.08) | 4.61 (±1.17) | 0.710 | 4.51 (±1.21) | 4.75 (±1.21) | 0.370 |
| Log Abundance (Prokaryotic Virus) | 3.18 (±1.51) | 3.54 (±1.11) | 0.630 | 3.54 (±1.45) | 3.25 (±1.00) | 0.620 | 3.22 (±1.28) | 3.54 (±1.35) | 0.500 | 3.17 (±1.31) | 3.47  (±1.32) | 0.500 |
| Log Abundance (Eukaryotic Virus) | 3.59 (±1.53) | 3.84 (±1.43) | 0.260 | 3.47 (±1.55) | 4.33 (±1.33) | 0.238 | 3.64 (±1.53) | 3.61 (±1.44) | 0.520 | 3.21 (±1.43) | 3.68 (±1.53) | 0.610 |
| Log Abundance (Non-Polio Eukaryotic Virus) | 2.94 (±1.80) | 2.87 (±1.93) | 0.550 | 3.08 (±1.88) | 2.83 (±1.87) | 0.710 | 2.96 (±1.92) | 2.8 (±1.82) | 0.860 | 2.87 (±1.86) | 2.94 (±1.88) | 0.820 |
| Log Abundance (Polio) | 0.36 (±1.62) | 1.34 (±1.98) | 0.130 | 0.57 (±1.66) | 1.84 (±2.04) | 0.084 | 0.94 (±1.88) | 0.79 (±1.88) | 0.780 | 0.9 (±1.62) | 0.48 (±2.00) | 0.580 |
|  |  |  |  |  |  |  |  |  |  |  |  |  |
| Chao Richness (Total) | 10  (±1.22) | 9 (±1.44) | 0.820 | 10  (±1.22) | 9 (±1.44) | 0.820 | 9 (±3.01) | 10 (±2.62) | 0.550 | 10 (±2.19) | 9 (±1.99) | 0.920 |
| Chao Richness (Prokaryotic Virus) | 4 (±1.38) | 5  (±1.44) | 0.900 | 4 (±1.57) | 5  (±1.44) | 1.000 | 4 (±1.17) | 5 (±1.30) | 0.850 | 4 (±1.53) | 4 (±1.45) | 0.430 |
| Chao Richness (Eukaryotic Virus) | 4 (±1.76) | 5 (±2.03) | 0.720 | 5 (±2.03) | 4 (±2.24) | 0.790 | 4.5 (±1.34) | 5 (±2.03) | 0.380 | 5 (±1.80) | 4 (±1.80) | 0.170 |
| Chao Richness (Non-polio Eukaryotic Virus) | 4 (±2.15) | 4 (±1.98) | 0.410 | 4 (±1.88) | 4 (±1.72) | 0.710 | 4 (±1.11) | 4 (±1.13) | 0.290 | 5 (±1.66) | 4 (±1.99) | 0.190 |
|  |  |  |  |  |  |  |  |  |  |  |  |  |
| Shannon Diversity (Total) | 0.45 (±0.38) | 0.58 (±0.43) | 0.520 | 0.52 (±0.44) | 0.53 (±0.67) | 0.910 | 0.59 (±0.70) | 0.43 (±0.35) | 0.300 | 0.57 (±0.35) | 0.45 (±0.23) | 0.530 |
| Shannon Diversity (Prokaryotic Virus) | 0.49 (±0.19) | 0.65 (±0.26) | 0.080 | 0.65 (±0.56) | 0.49 (±0.42) | 0.460 | 0.5 (±0.41) | 0.59 (±0.43) | 0.830 | 0.68 (±0.77) | 0.51 (±0.46) | 0.190 |
| Shannon Diversity (Eukaryotic Virus) | 0.18 (±0.20) | 0.3 (±0.13) | 0.810 | 0.25 (±0.30) | 0.20 (±0.38) | 0.920 | 0.19 (±0.20) | 0.25 (±0.33) | 0.760 | 0.47 (±0.24) | 0.38 (±0.29) | 0.370 |
| Shannon Diversity (Non-Polio Eukaryotic Virus) | 0.27 (±0.22) | 0.48 (±0.55) | 0.670 | 0.36 (±0.38) | 0.51 (±0.22) | 0.620 | 0.34 (±0.17) | 0.51 (±0.22) | 0.620 | 0.38 (±0.39) | 0.47 (±0.51) | 0.460 |

^a^ calculated using the Kruskal-Wallis rank sum test

**Supplementary Table S10. Poliovirus reads and associated antibody titers grouped by serotype in infant**

| Infant | Poliovirus, Sabin type 1 | | Poliovirus, Sabin type 2 | | Poliovirus, Sabin type 3 | |
| --- | --- | --- | --- | --- | --- | --- |
|  | RPM | NAb Titer (1/n)^a^ | RPM | NAb Titer (1/n) | RPM | NAb Titer (1/n) |
| 1 | 1 | −^b^ | 13 | 8 | 7 | − |
| 2 | 0 | − | 13 | − | 13 | 16 |
| 3 | 6 | 16 | 2 | 64 | 30 | − |
| 4 | 0 | − | 0 | − | 1 | 8 |
| 5 | 0 | 8 | 0 | 32 | 1 | − |
| 6 | 92,425 | 8 | 48,903 | 16 | 334 | 16 |
| 7 | 45,908 | 16 | 6,234 | 16 | 7 | 8 |
| 8 | 0 | − | 0 | 16 | 1 | 16 |
| 9 | 21 | 8 | 128 | 64 | 62 | 16 |
| 10 | 4 | − | 7 | 8 | 4 | − |
| 11 | 2 | 8 | 0 | 16 | 516,811 | − |
| 12 | 7,490,358 | 64 | 6,289,487 | 32 | 1,834,119 | 16 |
| 13 | 6 | − | 13 | 64 | 61 | − |
| 14 | 6 | − | 8 | 16 | 2 | 16 |
| PC^c^ | N/A | 8 | N/A | 8 | N/A | 8 |

^a^ Dilution at which 100% neutralization activity was observed

^b^ Samples that did not demonstrate neutralization at any titers tested

^c^ Positive control (PC) was serum from an individual with a history of inactivated poliovirus vaccination

Abbreviations: PC, positive control; Nab, neutralizing antibody; RPM, reads per million; −, negative by antibody neutralization

**Supplementary Table S11. Viral RPM and neutralizing antibodies detected against poliovirus**

| **Poliovirus RPM^a^** | **Log Polio RPM** | **Neutralizing Activity^b,c^** |
| --- | --- | --- |
| 6.61 | 0.82 | 2 |
| 4.54 | 0.66 | 2 |
| 0 | 0 | 2 |
| 0 | 0 | 3 |
| 0 | 0 | 2 |
| 0 | 0 | 3 |
| 0 | 0 | 3 |
| 0 | 0 | 2 |
| 0 | 0 | 1 |
| 2,816.25 | 3.45 | 3 |
| 7,391.52 | 3.87 | 1,2,3 |
| 3,163.07 | 3.50 | 1,2,3 |
| 24.49 | 1.39 | 1,2,3 |
| 833,475.30 | 5.92 | 1,2,3 |

^a^ median RPM for antibodies for all 3 Sabin types is 3.72log_10_ ± 1.86; median RPM for antibodies for 1 Sabin type only is 0.75log_10_ ± 0.41

^b^ neutralizing activity against Sabin strain(s) was defined as a dilution of ≥1:8

^c^ p=0.02 using the Mann-Whitney U test (comparison of neutralizing activity against RPM for all 3 Sabin types versus 1 Sabin type only)

**Supplementary Table S12. Virome associations with poliovirus serology status by stool collection time point**

|  | **Antibodies (TP1)** | | | **Antibodies (TP2)** | | | **Antibodies (TP3)** | | |
| --- | --- | --- | --- | --- | --- | --- | --- | --- | --- |
| **Virome Metric** | < 3 Sabin (n=10)  median (SD) | All 3 Sabin (n=4)  median (SD) | p-value^a^ | < 3 Sabin (n=10)  median (SD) | All 3 Sabin (n=4)  median (SD) | p-value^a^ | < 3 Sabin (n=10)  median (SD) | All 3 Sabin (n=4)  median (SD) | p-value^a^ |
| Log Abundance (Total) | 3.61 (±1.78) | 4.36 (±0.51) | 0.700 | 4.45 (±1.28) | 4.96 (±0.86) | 0.400 | 5.06 (±0.71) | 4.75 (±0.71) | 0.300 |
| Log Abundance (Prokaryotic Virus) | 2.48 (±1.77) | 2.2 (±1.81) | 0.900 | 2.97 (±1.40) | 3.39 (±0.44) | 0.600 | 2.96 (±0.92) | 2.97 (±0.53) | 1.000 |
| Log Abundance (Eukaryotic Virus) | 1.84 (±1.87) | 4.32 (±0.84) | 0.300 | 4.38 (±1.53) | 4.9 (±1.40) | 0.400 | 5.06 (±1.36) | 4.75 (±0.85) | 0.400 |
| Log Abundance (Non-Polio Eukaryotic Virus) | 1.52 (±2.02) | 4.19 (±0.79) | 0.300 | 2.68 (±2.07) | 2.19 (±2.47) | 0.900 | 5.06 (±1.92) | 4.48 (±1.14) | 0.400 |
| Log Abundance (Poliovirus) | 0 (±0.78) | 1.39 (±1.82) | 0.500 | 0.36 (±2.10) | 1.66 (±3.05) | 0.600 | 0 (±0.21)) | 0.97 (±1.04) | 0.090 |
|  |  |  |  |  |  |  |  |  |  |
| Chao Richness (Total) | 8 (±2.01) | 10 (±1.85) | 0.100 | 8 (±1.17) | 13 (±1.73) | 0.300 | 9 (±2.10) | 10 (±2.05) | 0.600 |
| Chao Richness (Prokaryotic Virus) | 3 (±1.68) | 6 (±1.54) | 0.200 | 4 (±1.65) | 6 (±2.00) | 0.20 | 3 (±1.63) | 5 (±1.92) | 0.200 |
| Chao Richness (Eukaryotic Virus) | 4 (±1.83) | 5 (±0.95) | 0.400 | 4 (±2.15) | 5 (±2.44) | 0.500 | 6 (±1.26) | 4 (±0.96) | 0.700 |
| Chao Richness (Non-Polio Eukaryotic Virus) | 4 (±1.83) | 5 (±1.81) | 0.600 | 4 (±3.06) | 4 (±3.17) | 0.900 | 6 (±0.19) | 3 (±2.45) | 0.600 |
|  |  |  |  |  |  |  |  |  |  |
| Shannon Diversity (Total) | 0.71 (±0.44) | 0.6 (±0.46) | 1.000 | 0.74 (±0.86) | 0.52 (±0.48) | 0.700 | 0.43 (±0.23) | 0.99 (±0.78) | 0.300 |
| Shannon Diversity (Prokaryotic Virus) | 0.53 (±0.45) | 1.02 (±0.79) | 0.100 | 0.58 (±0.78) | 1.07 (±1.28) | 0.30 | 0.34 (±0.53) | 1.07 (±0.46) | 0.300 |
| Shannon Diversity (Eukaryotic Virus) | 0.33 (±0.44) | 0.54 (±0.21) | 0.900 | 0.47 (±0.44) | 0.01 (±0.50) | 0.600 | 0.36 (±0.55) | 0.72 (±0.88) | 0.400 |
| Shannon Diversity (Non-Polio Eukaryotic Virus) | 0.39 (±0.34) | 0.54 (±0.28) | 1.000 | 0.56 (±0.27) | 0.49 (±0.31) | 1.000 | 0.36 (±0.59) | 0.52 (±0.35) | 0.600 |

^a^ calculated using the Kruskal-Wallis rank sum test

Abbreviations: TP:1, stool timepoint 1; TP2, stool timepoint 2; TP3, stool timepoint 3.

**Supplementary Table S13. Virome composition comparison of age-matched infants from Bangladesh and the United States.**

**ir**

| **Virome Metric** | **Bangladesh (n=30)**  **median (SD)** | **U.S.  (n=16)**  **median (SD)** | **p-value^a^** |
| --- | --- | --- | --- |
| Log Abundance (Total) | 4.69 (±0.99) | 3.0 (±1.28) | **0.004** |
| Log Abundance (Prokaryotic Virus) | 3.44 (±1.23) | 3.0 (±1.64) | 0.140 |
| Log Abundance (Eukaryotic Virus) | 4.22 (±1.28) | 1.5 (±0.64) | **<0.001** |
| Log Abundance (Non-Polio Eukaryotic Virus) | 3.43 (±1.60) | 1.5 (±0.64) | **0.003** |
| Log Abundance (Poliovirus) | 0.39 (±0.17) | 0 (±0) | **<0.001** |
|  |  |  |  |
| Chao Richness (Total) | 9 (±2.05) | 6 (±1.42) | **0.010** |
| Chao Richness (Prokaryotic Virus) | 4 (±1.75) | 3.5 (±1.88) | 0.098 |
| Chao Richness (Eukaryotic Virus) | 4 (±1.06) | 2 (±0,83) | **0.003** |
| Chao Richness (Non-Polio Eukaryotic Virus) | 4 (±1.89) | 2 (±0.57) | **0.005** |
|  |  |  |  |
| Shannon Diversity (Total) | 0.49 (±0.28) | 0.58 (±0.19) | 0.700 |
| Shannon Diversity (Prokaryotic Virus) | 0.51 (±0.39) | 0.31 (±0.22) | 0.270 |
| Shannon Diversity (Eukaryotic Virus) | 0.25 (±0.19) | 0.17 (±0.07) | 0.190 |
| Shannon Diversity (Non-Polio Eukaryotic Virus) | 0.35 (±0.09) | 0.17 (±0.11) | 0.200 |

^a^ comparison of Bangladeshi versus US cohorts was performed using PERMANOVA with the Adonis Function (1000 permutations)

**REFERENCES**

1 Benjamini, Y. & Hochberg, Y. Controlling the False Discovery Rate - a Practical and Powerful Approach to Multiple Testing. *Journal of the Royal Statistical Society Series B-Statistical Methodology* **57**, 289-300 (1995).
